# Bio-psycho-social medicine is a comprehensive form of medicine bridging clinical medicine and public health

**DOI:** 10.1186/1751-0759-4-19

**Published:** 2010-11-05

**Authors:** Mutsuhiro Nakao

**Affiliations:** 1 Department of Hygiene and Public Health, Teikyo University School of Medicine, Tokyo, Japan; 2 Division of Psychosomatic Medicine, Teikyo University Hospital, Tokyo, Japan

## 

The journal, *BioPsychoSocial Medicine*, was launched in January 2007, and more than three and a half years have passed since the start of publication. A total of 70 articles have been published, as of August 2010, but the number of those directly discussing issues of public health or social medicine is relatively small [1-4]. Psychosomatic medicine, or biopsychosocial medicine, encompasses all aspects of the interrelationships between the biological, psychological, social, and behavioral factors of health and illness, and is not limited to mind and body connections in humans. Thus, it is meaningful that social aspects of health and illness are discussed in the current issue of this journal.

In the symposium addressing the "healthy hospital", Kawachi has set the scene for a debate about people's health by pitting medical care against population-level preventive activities [5]. In this debate, patient-centered clinical care is positioned at one pole, while population-wide public health activities - such as encouraging healthier lifestyles or improving the physical and social environment - end up being positioned at the opposite pole. Such debates are unnecessarily polarizing and run the risk of oversimplifying the determinants of population health to the point of caricature [5]. According to Article 1 of the Medical Practitioners Act in Japan [6], medical practitioners or physicians should focus on the improvement and promotion of public health by using both medical care and health guidance to secure a generally healthy life for a broad range of people in Japan. Clinical medicine is closely linked to a public health approach, and medical practices should be undertaken within the limited human, time, and financial resources available. In addition to a high-risk approach for those who suffer or are likely to suffer from illnesses, a population approach emphasizing health communication, a health program, and the health system should be employed to maintain people's health. In this sense, psychosomatic medicine helps to create a bridge between clinical medicine and public health.

In this special series on public health, five articles are presented to introduce important concepts of social medicine relevant to the research fields of psychosomatic medicine. The first concept is health literacy [7]. Health literacy is a concept that has been intensively applied to medical research since the 1990s and defined as the cognitive and social skills that determine the motivation and ability of individuals to gain access to, understand, and use information in ways that promote and maintain good health [8]. Ishikawa and her colleagues have been studying health literacy after publishing several reports examining the patient-doctor relationship [9-11]. Communication between patients and physicians is fundamental for improved patient care, but physicians often ignore or are not focused on levels of patient knowledge. With increasing medical costs, self-care, including the early recognition of disease, has become increasingly important as a way of reducing medical expenditures. Health literacy should be developed at both the individual and mass media levels for appropriate medical care.

The second concept is work engagement [12]. Burnout syndrome due to overwork has been intensively studied for many years, but this approach is ill-health oriented. Occupational health psychology involves the application of psychology to improving the quality of work life and to protecting and promoting the safety, health, and well-being of workers [13]. Engagement is defined as a positive, fulfilling, work-related state of mind that is characterized by vigor, dedication, and absorption. Shimazu and his colleagues have focused on work engagement in occupational health psychology [13-15], and their intensive studies should lead to the development of a positive psychology and better management of job stress [16].

Third, the concept of effort-reward imbalance and the practical issue of job insecurity are discussed in association with job stress [17]. Work-related stress includes the factors of job stress, employment status, job insecurity, and lack of work-family balance [4]. Among these factors, job stress is a substantial and growing concern for workers, their advocates, employers, occupational health and safety regulators, and workers' compensation programs, and has been linked to a range of adverse physical and mental health outcomes, including cardiovascular disease, insomnia, depression, and anxiety. The job demand-control-support model proposed by Karasek and Theorell [18] is relatively common in the field of occupational health, and the effort-reward imbalance model developed by Siegrist [19] has also been used for job-stress research. In this model, high-cost and low-gain work conditions are particularly stressful. Concerning job insecurity, temporary or term-limited workers often complain that they are more productive but receive lower compensation than permanent workers. Our own research has found that term-limited workers tend to work more hours and experience symptoms of fatigue more frequently than do permanent workers [20]. However, current working conditions have become relatively complex; for example, Inoue and her colleagues have shown that permanent and temporary workers suffer from different aspects of job stress [17].

The fourth concept under examination is health risk assessment and management, for which depression and suicide problems among workers can serve as an example [21]. Depression constitutes one of the leading causes of disability in the world and is commonly encountered in primary care settings, in the workplace and school, and in the community [22]. Upon identification, depressed patients need appropriate management, including pharmacological treatment, sufficient rest, and social support. Depression clearly plays an important role in the epidemiology of suicide [23]; more than 60% of individuals who commit suicide are depressed, and identifying individuals at high risk for depression might reduce the number of suicide attempts. In response to epidemics of depression and suicide, Takeuchi and his colleagues have been researching depression screening [24-26], and they have developed a method of matrix assessment for depression in the workplace, categorizing the working environment, working conditions, and workers' health as three factors at three different levels of primary, secondary, and tertiary prevention.

Fifth, quality assessment of care is discussed [27]. Evidence-based quality indicators have increasingly been used to evaluate medical providers and to promote both transparency of care and competition based on quality [28]. In the United States, for example, reimbursement is linked to performance measured with the use of quality metrics in pay-for-performance programs. However, the Japanese health care system guarantees equal access for equal needs with universal insurance coverage. According to Siegrist's theory [29], the quality of health care should be measured in terms of the three pillars of quality performance: outcome measures (e.g., mortality, complications), process measures (e.g., clinical compliance in specific diseases, operational compliance such as waiting times and bed placement), and satisfaction measures (e.g., feedback from patients and physicians, nurses, and staff). Higashi continues his research, in this regard, assessing the quality of care [30-32].

Through these five articles we can learn much about the need to incorporate ideas pertaining to the field of public health into clinical medicine to form a new discipline uniting the two areas. Psychosomatic medicine practitioners and researchers are well qualified to bring about this union.

## Competing interests

The author has no competing interests to declare.

## Authors' contributions

The author wrote the manuscript and holds final responsibility for the decision to submit the manuscript for publication.
